# Virus-Like Particle (VLP) Plus Microcrystalline Tyrosine (MCT) Adjuvants Enhance Vaccine Efficacy Improving T and B Cell Immunogenicity and Protection against *Plasmodium* *berghei/vivax*

**DOI:** 10.3390/vaccines5020010

**Published:** 2017-05-02

**Authors:** Gustavo Cabral-Miranda, Matthew D. Heath, Mona O. Mohsen, Ariane C. Gomes, Paul Engeroff, Amy Flaxman, Fabiana M. S. Leoratti, Aadil El-Turabi, Arturo Reyes-Sandoval, Murray A. Skinner, Matthias F. Kramer, Martin F. Bachmann

**Affiliations:** 1 The Jenner Institute, Nuffield Department of Medicine, Centre for Cellular and Molecular Physiology (CCMP), University of Oxford, Oxford OX3 7BN, UK; mona.mohsen@kellogg.ox.ac.uk (M.O.M.); ariane.cruzgomes@ndm.ox.ac.uk (A.C.G.); amy.flaxman@ndm.ox.ac.uk (A.F.); aadil.el-turabi@ndm.ox.ac.uk (A.E.-T.); arturo.reyes@ndm.ox.ac.uk (A.R.-S.); 2 Allergy Therapeutics (UK) Ltd. Dominion Way, Worthing BN14 8SA, UK; Matthew.Heath@allergytherapeutics.com (M.D.H.); Murray.Skinner@allergytherapeutics.com (M.A.S.); 3 Immunology, RIA, Inselspital, University of Bern, Bern 3012, Switzerland; paul.engeroff@students.unibe.ch (P.E.); fabiana.leoratti@gmail.com (F.M.S.L.); 4 Bencard Allergie, Messerschmittstraße 4, München 80992, Germany; KramerM@bencard.com

**Keywords:** vaccine, adjuvants, *Plasmodium vivax*, malaria, virus like particle (VLP), microcrystalline tyrosine (MCT)

## Abstract

Vaccination is the most effective prophylactic tool against infectious diseases. Despite continued efforts to control malaria, the disease still generally represents a significant unmet medical need. Microcrystalline tyrosine (MCT) is a well described depot used in licensed allergy immunotherapy products and in clinical development. However, its proof of concept in prophylactic vaccines has only recently been explored. MCT has never been used in combination with virus-like particles (VLPs), which are considered to be one of the most potent inducers of cellular and humoral immune responses in mice and humans. In the current study we assessed the potential of MCT to serve as an adjuvant in the development of a vaccine against malaria either alone or combined with VLP using *Plasmodium vivax* thrombospondin-related adhesive protein (TRAP) as a target antigen. We chemically coupled PvTRAP to VLPs derived from the cucumber mosaic virus fused to a universal T-cell epitope of the tetanus toxin (CMVtt), formulated with MCT and compared the induced immune responses to PvTRAP formulated in PBS or Alum. The protective capacity of the various formulations was assessed using *Plasmodium berghei* expressing PvTRAP. All vaccine formulations using adjuvants and/or VLP increased humoral immunogenicity for PvTRAP compared to the antigen alone. The most proficient responder was the group of mice immunized with the vaccine formulated with PvTRAP-VLP + MCT. The VLP-based vaccine formulated in MCT also induced the strongest T cell response and conferred best protection against challenge with recombinant *Plasmodium berghei*. Thus, the combination of VLP with MCT may take advantage of the properties of each component and appears to be an alternative biodegradable depot adjuvant for development of novel prophylactic vaccines.

## 1. Introduction

Malaria is a massive global health problem [1,2,3]. The World Health Organization (WHO), estimated in December 2015 the occurrence of 214 million cases and 438,000 deaths caused by malaria worldwide [4]. Although five parasite species exist that cause malaria in humans, two of them appear to be the most dangerous: *Plasmodium falciparum* and *Plasmodium vivax* [5,6]. Despite being a threat for decades, to date there is no licensed malaria vaccine for human use. Only one vaccine against *P. falciparum* (RTS,S/AS01) is undergoing licensure as it has shown promising, albeit limited, efficacy in clinical trials [7]. However, there is no vaccine for *P. vivax* in late-stage development, although this parasite is most prevalent in most countries outside the African continent.

Many antigens have been tested in vaccine development, and a promising malaria vaccine candidate is the thrombospondin-related adhesive protein (TRAP), a transmembrane protein with extracellular adhesive domains essential for sporozoite motility and liver cell invasion [8]. TRAP has been reported to be a target for T cell-based vaccines, but some studies have also shown the importance of antibodies against TRAP as protective effector molecules against malaria [9,10,11].

Besides selecting an effective antigen, a second important component for vaccine development is the use of an adjuvant. Adjuvants can modulate the immune response against specific antigens and enhance immunogenicity [12]. Since the 1920s, when the first adjuvant (aluminium salts) was used in humans, the concept of adjuvants has become a key target for improving the efficacy of modern vaccines [13]. Aluminium-based preparations remain the most commonly used adjuvants in both human and veterinary vaccines [14,15].

Aluminium-based adjuvants have a safety record and positive benefit risk profile in the context of prophylactic vaccination programmes. Aluminium hydroxide adjuvants (alum) is cost-effective, easy to manufacture and licensure of alum formulated products is comparably straight-forward. As a result, its major use is somewhat habitual rather than tailored and rationally selected for its intended specific application. Therefore, it does not come without its limitations when addressing more complex pathogen-associated immunological challenges and associated unmet needs. For example, alum will not necessarily provide an optimal choice in supporting the immunological effect of a specific indication that requires development of Th1/adaptive responses [15,16,17,18,19]. In addition to this, alum has no biological function, is non-biodegradable and has been associated with Th2 responses which promote secretion of proinflammatory cytokines such as interleukin (IL)-1 and cell apoptosis [14,20,21]. These disadvantages encourage the use of natural and biodegradable alternative platforms that can support the immunological effect of a specific therapy.

Microcrystalline tyrosine (MCT) is one such alternative candidate. MCT is a depot adsorbant-base that has been successfully used in allergen-specific immunotherapy for the treatment of allergy for a number of years [22]. MCT has previously shown strong capacity to induce IgG antibodies, develop Th1-biased responses and increase protection against malaria when compared with alum adjuvants [22]. The mechanism of action of MCT is currently being studied. MCT has been shown to trigger stronger IFN-γ and IL-10 production in spleen cells than immunisation in the presence of alum. Measurements investigating specific T cell responses, dendritic cell (DC) activation and expression markers in challenge models are still ongoing and previous results from these ongoing pre-clinical immunogenicity studies in mice have demonstrated the comparative potential of MCT with alum in stimulating antibody responses and T-cell responses [23]. The favourable Th1-like immune responses produced by MCT suggest that MCT may meet the requirements for a wide range of future vaccines and immunotherapies. As MCT potentially exhibits physicochemical and immunological advantages over alum, there is rationale to explore its potential in developing vaccines against infectious diseases, such as malaria.

Virus-like particles (VLPs) have a high capacity to induce strong humoral and cellular immune responses [24,25,26] and may have the potential to increase vaccine efficacy against malaria in particular if combined with MCT to create a synergistic adjuvant system. Besides strong immune-stimulatory capacity, VLPs share some characteristics with MCT which are both natural components recognised by the host with the propensity to initiate a Th1-biased innate response and prime adaptive mechanisms.

In the current study, we assessed the ability of MCT to enhance the immunogenicity of *Plasmodium vivax* thrombospondin-related adhesive protein (PvTRAP) in free form or conjugated to VLPs and to protect against infection with *Plasmodium bergei* expressing *P. vivax* TRAP [27].

## 2. Material and Methods

### 2.1. Construction, Expression and Purifications of PvTRAP Protein

Previously, PvTRAP was synthesised by Geneart™, then it was cloned to make viral-vectored vaccines [27], and it was also sub cloned into expression vector pHLsec to produce our protein with a C-terminal histidine tag fusion. A set of primers from the conserved sequences, one near the putative signal peptide cleavage site: forward primer (5′ taaaccggtGACGAGAAGGTGGTGGACG 3′), and the other at the extreme carboxyl-terminus: reverse primer (5′ attggtaccCTTGTAGCCGTTGTTGCTGG 3′) was designed and synthesized by Sigma-Aldrich. PCR amplification was achieved using *P. vivax* genomic DNA and the product was cloned into the pHLsec vector at AgeI and KpnI restriction sites. The new plasmid obtained (pHLsec-PvTRAP) was transfected into the bacteria DH5a for production at a large scale. The protein was expressed by transient transfection in HEK-293T cells (Sigma-Aldrich, Gillingham, UK) and purified from dialyzed conditioned medium against phosphate-buffered saline (PBS) buffer by immobilized nickel affinity chromatography, followed by size exclusion chromatography in 20 mM Tris-HCl, pH 8.0, and 300 mM NaCl.

### 2.2. Vaccines Preparation Based on PvTRAP Protein

#### 2.2.1. CMVtt-PvTRAP (Chemically Coupled) Vaccine Preparation

The Phyto-VLP construct encoding the coat protein of cucumber mosaic virus fused to a universal T-cell epitope of the tetanus toxin (CMVtt) will be described in detail elsewhere (A. Zeltins, in preparation). VLPs were expressed in *Escherichia coli* BL21 (DE3) Star (Thermo Fisher Scientific, Loughborough, UK) and purified from the soluble fraction of cell lysates by repeated precipitation with 40% (v/v) PEG8000 (Sigma-Aldrich, Gillingham, UK) and ammonium sulphate solution to yield soluble particles that were further purified by size exclusion chromatography (16/600 Sephacryl S-500HR, GE Healthcare, Life Sciences, Amersham, UK). The PvTRAP protein was covalently conjugated to CMVtt by a two-step procedure. First, CMVtt VLP at 1 mg/mL in 20 mM sodium phosphate, 2 mM EDTA, and 30% (w/v) sucrose, at pH 7.2 (PES buffer), were reacted at room temperature for 30 min with a 10-fold molar excess of the heterobifunctional chemical cross-linker, succinimidyl-6-(b-maleimidopropionamido) hexanoate (SMPH) (Thermo Fisher Scientific). Unreacted SMPH cross-linker was removed by diafiltration against PES buffer using 100 kDa Amicon Ultra centrifugal filters (Millipore, Billerica, MA, USA). Prior to the conjugation step, purified PvTRAP was incubated for 30 min at room temperature with a 6-fold molar excess of N-succinimidyl-S-acetylthioacetate (SATA) (Thermo Fisher Scientific); excess SATA cross-linker was removed by diafiltration as before using 3 kDa Amicon Ultra centrifugal filters (Millipore) and derivatised protein was then de-protected using hydroxylamine (3 h at room temperature) resulting in the addition of reactive sulphydryl residues to the protein. Following a further diafiltration, PvTRAP-SATA was covalently linked to the derivatised CMVtt by reacting equimolar amounts of PvTRAP-SATA and CMVtt-SMPH for 4 h at room temperature (RT). The conjugated pVLP vaccine was analysed by SDS-PAGE and western blot to detect bands corresponding to the various components of the coupling reaction.

#### 2.2.2. Vaccine Formulation Using MCT as Adjuvant

The formulation was prepared based on Allergy Therapeutics’ experience in allergen formulation. MCT was used at 2% (20 mg/mL) diluted with PBS and the antigen (PvTRAP or CMVttVLP-PvTRAP) was added and mixed well. The injection was administered immediately after the vaccine preparation.

#### 2.2.3. Vaccine Based on Aluminium Hydroxide Adjuvant (Alum)

The vaccine formulation was done by adding 10 µg of Al3+ per dose at 50 mL per mouse and prepared according to the manufacturer’s specifications (InvivoGen, San Diego, CA, USA). Briefly, the alum was mixed with 20 mM TRIS buffer (pH 7.0–7.5) and left at RT for 15 min. Subsequently the antigen/protein was added to the corresponding Eppendorf tubes containing alum + TRIS buffer and vortexed gently for five seconds. After that, the vaccine was incubated at RT for one hour and vaccination occurred as soon as possible within the same day.

### 2.3. Experimental Design

**Figure 1:**
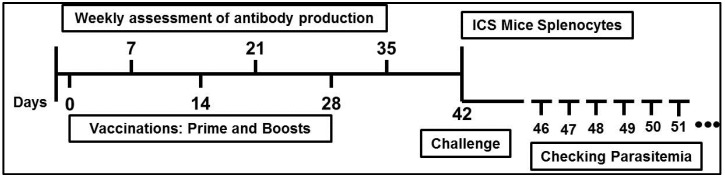
Experimental design using *Plasmodium vivax* thrombospondin-related adhesive protein (PvTRAP) protein as antigen: the schedule was designed to assess the kinetics of antibody production; T cell responses and protection against *Plasmodium berghei* expressing PvTRAP. BALB/c mice were vaccinated intramuscularly three times: prime (Day 0), first boost (Day 14) and second boost (Day 28). Serum was taken weekly until one week before the challenge. On Day 42, 5 mice per group were culled to collect the spleens for intracellular cytokine staining (ICS), and another 6 were challenged with *P. berghei* exhibiting PvTRAP replacement. Parasitaemia was checked daily, from the fourth day after challenge until the mice reached 1% parasitaemia.

In order to assess the humoral immunity, T cell response, and protection against *Plasmodium bergei* replacement expressing *P. vivax* TRAP, eleven female BALB/c mice (age-matched, 6 weeks old, purchased from Harlan, United Kingdom) were vaccinated intramuscularly (i.m) with 10 µg (50 μL) of PvTRAP protein chemically coupled with VLP with or without MCT; protein plus alum or MCT adjuvants; or only protein in PBS. The vaccinations were done 3 times: prime (Day 0), first boost (Day 14) and second boost (Day 28) (Figure 1). The samples (sera) were weekly collected, starting before the first vaccination (Day 0) up to Day 35 (one week before the challenge). On Day 42, 5 mice per group were culled and splenocytes were used for detection of IFN-γ and TNFα by intracellular cytokine staining (ICS). Six mice per group were challenged with *P. berghei* PvTRAP replacement and parasitaemia was checked daily beginning on fourth day after the challenge until the mice reached 1% parasitaemia.

### 2.4. Antibody Production Assessment

Enzyme-linked immunosorbent assays (ELISAs) were carried out to measure total and subclasses of IgG. Primary, 96-well microtitre ELISA plates (Thermo Scientific, Nottingham, UK) were coated with 100 µL per well at 1 µg/mL of PvTRAP diluted in carbonate buffer (CBB) 50 mM at pH = 9.6 and incubated overnight at 4 °C. To avoid nonspecific binding, the wells were filled with 200 μL of blocking solution (10% skim milk powder (Sigma-Aldrich, Irvine, UK) in PBS with 0.05% tween 20) and let stand at room temperature for 2 h. After that, serial dilutions were made starting at 1 in 100, followed by eleven 1/3 serial dilution in 0.25% BSA-PBS buffer in ELISA plate. Plate-bound antigen was saturated with antibody in sera to the lowest sera dilution until it became too low to be detected. For whole IgG assessment, 50 μL per well of detecting antibody (Ab) was added to goat anti-mouse IgG diluted to 1:2000 (Secondary Antibody, HRP conjugate (ThermoFisher, Paisley, UK), and incubated for 1 h at RT. For detection of IgG subclasses, subclass-specific secondary antibodies were used, diluted to 1:3000 (goat anti-mouse IgG1, IgG2a, IgG2b, IgG3, HRP coupled, Life Technologies). Titres are expressed as dilutions leading to half-maximal OD (OD_50_).

### 2.5. Intracellular Cytokine Staining (ICS)

The analysis of T cell response was done by ICS, detecting IFN-γ and TNF-α production. Spleens were collected from vaccinated and naïve mice, disrupted and red blood cells lysed with ammonium-chloride-potassium (ACK) solution (Lonza, Basel, Switzerland). A total of 1 × 10^6^ splenocytes were stimulated for 12 h in the presence of 1 µg PvTRAP-specific peptide (ITKVIPMLNGLINSLSLSRD) for T cell responses with immunodominant epitope [27]. In order to increase the accumulation of cytokines in the Golgi complex and enhance the capability of stain the cytokines produced, brefeldin A (BFA) (BD GolgiPlug™) was also added and left for 12 h, simultaneously with the peptide. To determine the cell viability, aqua live/dead cell staining (Thermo Fisher Scientific) was added to cells before fixation, permeabilization and cytokine staining. Functional CD3+ T cells were stained using the antibodies Anti-Mouse CD3 Alexa Fluor^®^ 700 (Clone: 17A2), Anti-Mouse Tumor Necrosis Factor alpha (TNF-a) APC (Clone: MP6-XT22) and Anti-Mouse Interferon-gamma (IFN-g) (Clone: XMG1.2). Flow cytometry assessment was carried out using a BD FACSCanto™ Flow Cytometer (BD Biosciences, Oxford, UK). Data were analysed with either FlowJo (Flowjo, Ashland, OR, USA) or GraphPad Prism (Graphpad Software, Inc., San Diego, CA, USA) software applied to assess the means by one-way analysis of variance (one-way ANOVA) and an unpaired t test was employed to compare two normally distributed groups.

### 2.6. Parasite Production and Challenge

The construction of the *P. bergei* replacement expressing *P.vivax* TRAP used for challenge in this experiment has been previously fully described [27]. In summary, the parasite chosen to evaluate the vaccine efficacy is a transgenic *P. berghei* in which the endogenous PbTRAP gene was irreversible, replaced with its orthologue from *P. vivax*. Furthermore, this transgenic *P. berghei* replacement PvTRAP was exhaustively tested in terms of infection capacity in a vertebrate host to infect the liver and develop within mosquitoes; as previously described it did not diminish the parasite’s infection ability compared to wild-type *P. bergei* parasite [27]. This parasite was obtained from the insectary of the Jenner Institute, University of Oxford, UK, where female *Anopheles stephensi* mosquitoes were fed using infected Tuck-ordinary (TO) inbred mice. For that, mosquitoes were exposed to anesthetized infected TO mice and then maintained for 3 weeks in a humidified incubator. Thereafter, salivary mosquitoes’ glands were dissected and 1000 parasites (100 µL) were intravenously injected into the tail vein of each mouse.

### 2.7. Statistical Model Applied for Predict Parasitaemia and Protection

The assessment of liver-to-blood parasitaemia and prediction of blood-stage parasite infection was done as previously described [28]. In summary, blood smears of each mouse were stained with Giemsa and 1000 red cells were counted. The parasitaemia was calculated in all mice and survival analysis was done using GraphPad Prism software to assess vaccine efficacy using time to reach 1% parasitaemia.

### 2.8. Ethics Statement

All animals and procedures were used in accordance with the terms of the United Kingdom Home Office and under regulation of The Animals (Scientific Procedures) Act 1986 [29]. The Project License was approved by the University of Oxford Animal Care and Ethical Review Committee (PPL 30/2947). The mice were housed in ventilated cages, under specific pathogen-free conditions, constant temperature, humidity and with a 12:12 light-dark cycle. For induction of short-term anaesthesia, mice were anaesthetized using vaporized IsoFlo. All animals were humanely sacrificed at the end of each experiment by an approved Schedule 1 method (cervical dislocation).

## 3. Results and Discussion

### 3.1. Generation of Vaccine Candidates and Formulation of VLPs with MCT

The protein was expressed by HEK-293T (Thermo Fisher Scientific) transfected with a plasmid encoding His-tagged PvTRAP. The HEK 293T cell supernatant was purified with a Ni-column followed by size exclusion chromatography. The purified protein was submitted to SDS-PAGE and proteins were visualized using coomassie blue and silver staining. The purified protein was identified using anti His-tag antibodies (6x-His Tag Antibody (HIS.H8)—Thermo Fisher Scientific). As predicted, the PvTRAP had a molecular weight of about 70 kDa. The elution samples were concentrated for the vaccine preparation and immunoassay.

**Figure 2:**
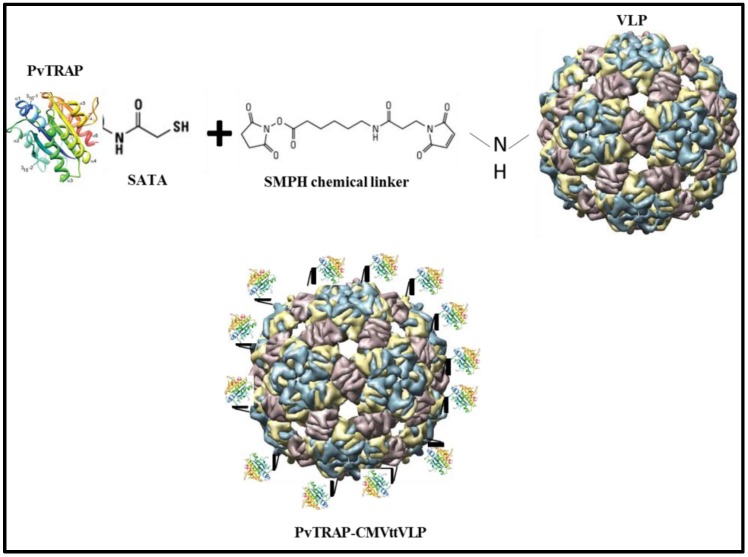
Vaccine preparation scheme: The conjugation of PvTRAP to CMVtt was done by modifying the CMVttVLP with a chemical cross-linker (SMPH) and binding it to modified PvTRAT protein with sulphydryl groups (-SH). SATA: N-succinimidyl-S-acetylthioacetate; VLP: virus-like particle.

The VLP-based vaccine preparation was made by covalently conjugating PvTRAP protein to CMVtt (The construction and properties of these VLPs will be described in detail elsewhere). For that, CMVttVLP first reacted with a chemical cross-linker, SMPH, which reacts to Lys on the VLP and can further be reacted to free sulphydryl groups (-SH) on the antigen (Figure 2). To this end, SH groups were added to the protein using SATA, as PvTRAP has no free Cys groups.

**Figure 3:**
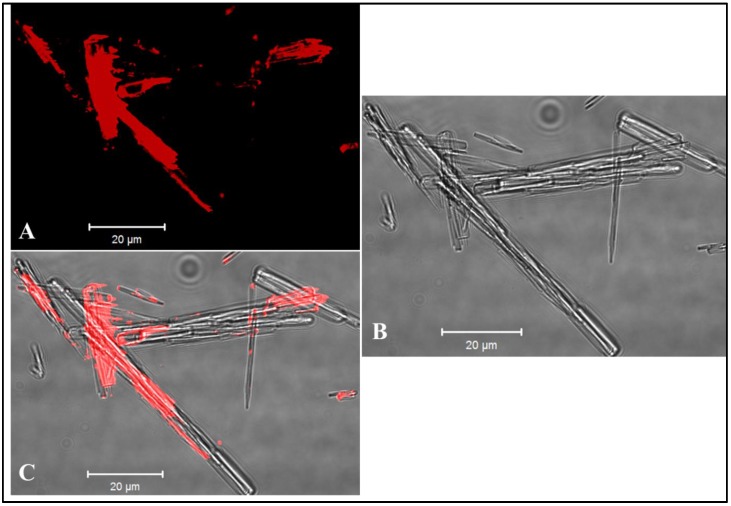
CMVtt CMV was labelled with Alexa Fluor 488 and formulated with microcrystalline tyrosine (MCT). Crystals were analysed by confocal microscopy. (**A**) Fluorescence of CMVttVLP; (**B**) bright field; and (**C**) Overlay.

MCT has been formulated with numerous proteins [30,31], but to date has not been formulated with VLPs. In order to visualize VLP-absorbance to MCT, we labelled CMVtt VLPs with Alexa Fluor 488 (Alexa Fluor^®^ 488 Protein Labeling Kit, Thermo Fisher Scientific), formulated with MCT and analysed the MCT crystals by confocal microscopy. As previously seen for proteins, VLPs bind to MCT crystals and decorate their surface (Figure 3).

### 3.2. Humoral Immune Responses

**Figure 4:**
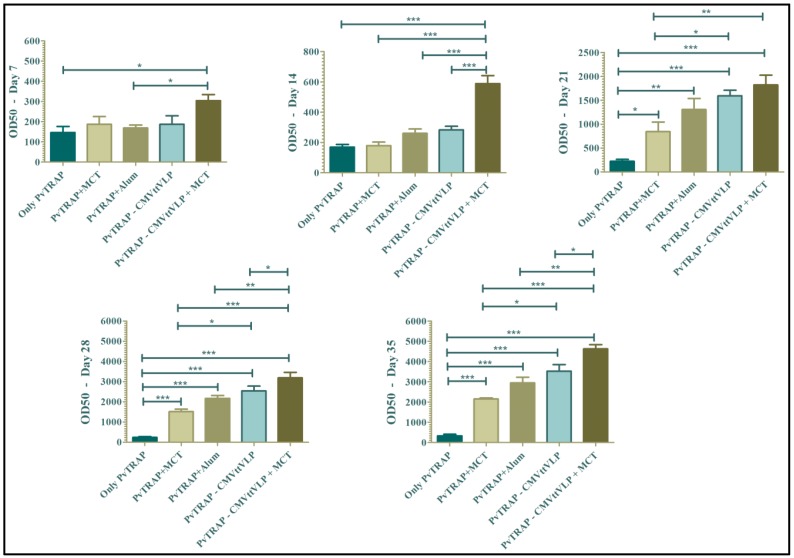
Assessment of kinetics of antibody production: the figure shows IgG total of all groups of mice bled weekly, from Day 7 (one week after the first vaccination) to Day 35 (one week after the second boost). The results were analysed using GraphPad Prism software applied to assess the means of five groups by one-way analysis of variance (one-way ANOVA). The values observed in the negative control group were subtracted from the titres of the other experimental groups. Note: *** = *p* value < 0.0001; ** = *p* value < 0.001; * = *p* value < 0.01.

Specific antibodies are crucial for protection against malaria and it has been shown in experimental animals [32,33,34] as well as in studies in endemic area [35,36,37], that natural humoral immunity to malaria is highly associated with protection. Furthermore, monoclonal antibodies are considered as novel weapons against the disease [38,39]. Even though TRAP may primarily induce protective T cells, antibodies have also been shown to be involved in protection. For these reasons, we assessed the kinetics of antibody production, quantifying specific IgG weekly. As shown in Figure 4, the group vaccinated with PvTRAP-CMVttVLP + MCT mounted the highest antibody response at all time points. The group vaccinated with PvTRAP-CMVttVLP in absence of MCT also mounted better responses than mice immunized with free TRAP formulated in adjuvants. TRAP formulated in alum induced slightly higher responses than TRAP formulated in MCT; however, there was no statistically significant difference between the groups at any time point. PvTRAP-CMVttVLP + MCT generated higher antibody titres which were detected earlier than the other experimental groups after a single vaccination at Day 14 (*p* ≤ 0.0001) and responses remained higher until the final bleed on Day 35.

**Figure 5:**
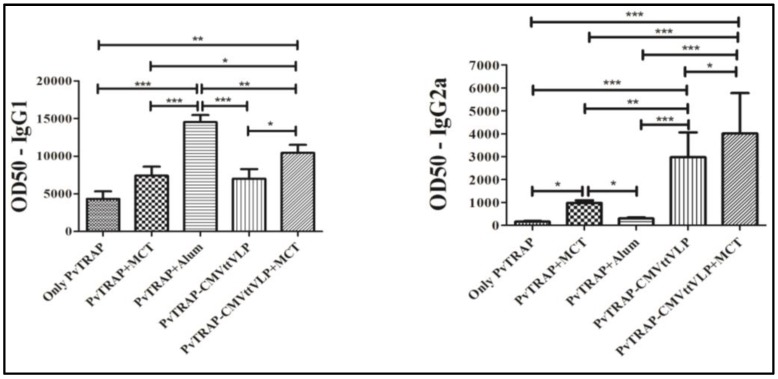

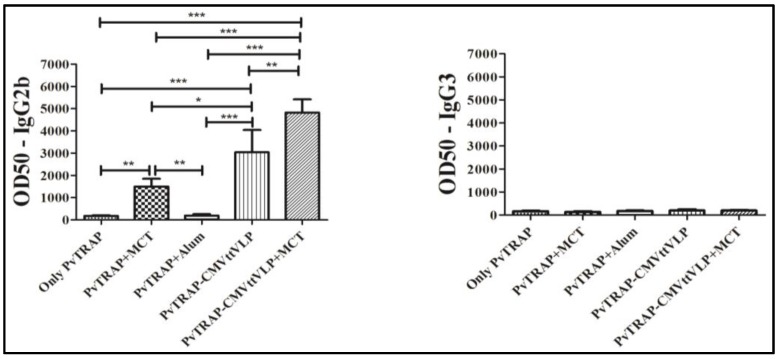
Analysis of relevant subclasses of IgG (IgG1, IgG2a, IgG2b, IgG3). In order to check the prevalent subclass of IgG, enzyme-linked immunosorbent assay (ELISA) was done using samples of all vaccinated groups on Day 35 (one week before challenge). The results were analysed using GraphPad Prism software applied to assess the means of five groups by one-way analysis of variance (one-way ANOVA). The values observed in the negative control group were subtracted from the titres of the other experimental groups. Note: *** = *p* value < 0.0001; ** = *p* value < 0.001; * = *p* value < 0.01.

IgG subsets were assessed using samples from Day 35, one week before the challenge. The dominant subclass in the group vaccinated with antigen-only was IgG1 and this antibody subset also appeared in all other groups on all days. The two groups vaccinated with protein coupled with CMVttVLP with or without MCT significantly induced IgG2a and IgG2b (Figure 5) with high and sustained antibody levels. Regarding the groups immunized with vaccine containing alum or MCT adjuvants alone, only the MCT group was able to significantly increase levels of other subclasses other than IgG1, namely IgG2a and IgG2b. Alum maintained IgG1 as the dominant subset of IgG. The literature shows that IgG2a is the most protective subclass associated with protection against *Plasmodium* infection [40,41,42]. Other IgG subclasses, as e.g., IgG3 in humans (usually associated with IgG2b in mice) have been significantly associated with protection against malaria in humans [36,41,43], and high levels of IgG2b in mice may also contribute to protective efficacy [40,44]. This therefore shows the importance of IgG subclasses in our results, especially for those groups of mice vaccinated with VLP and/or MCT.

### 3.3. Cellular Immunogenicity

**Figure 6:**
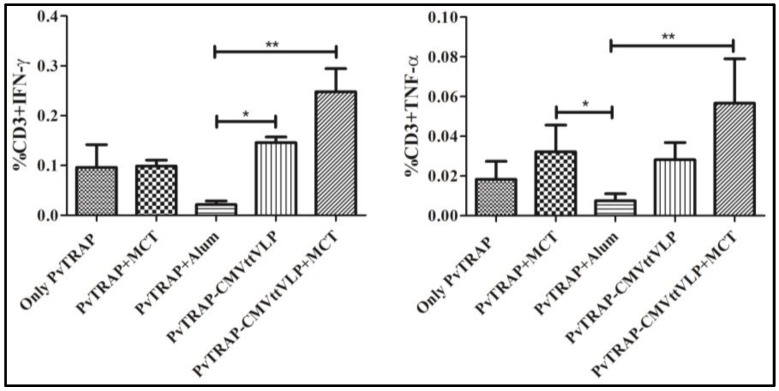
Analysis of T cell response by detecting IFN-γ and TNF-α. The frequencies of IFN-γ and TNF-α T cell responders were quantified by ICS as previously described, using splenocyte stimulated for 12 h in the presence of PvTRAP-specific peptide. The graphs show means and standard errors of the mean (SEM) of five mice per group. Data were analysed with either FlowJo or GraphPad Prism software applied to assess the means of five groups by one-way analysis of variance (one-way ANOVA); and an unpaired *t test* was employed to compare two normally distributed groups. **, *p* value < 0.001; *, *p* value < 0.01.

One of the goals of vaccine development for malaria is not only to induce high titre of antibodies but also to induce protective cellular immunity [45,46]. TRAP is a major target for T cell-based vaccines [46,47,48]. For this reason, T cell responses induced by PvTRAP coupled to VLP with or without adjuvant were also measured, by detecting IFN-γ and TNF-α which are important immunological mediators that can play a protective function in malaria [47,49]. Production of IFN-γ and TNF-α during T cell response was quantified by ICS upon stimulation with a PvTRAP-specific MHC class I restricted peptide (ITKVIPMLNGLINSLSLSRD) specifically inducing CD8^+^ T cells as previously described [27]. As shown in Figure 6, the highest percentage of IFN-γ and TNF-α secreting T cells were induced in mice immunized with PvTRAP-CMVttVLP + MCT. Responses induced by PvTRAP + MCT were similar to the responses induced PvTRAP-CMVttVLP or PvTRAP alone. A lower percentage response in the group vaccinated with PvTRAP + Alum was noted compared to all other groups, indicating that alum may not be a good inducer of CD8^+^ cytotoxic T cells.

### 3.4. Assessment of Vaccine Efficacy

**Figure 7:**
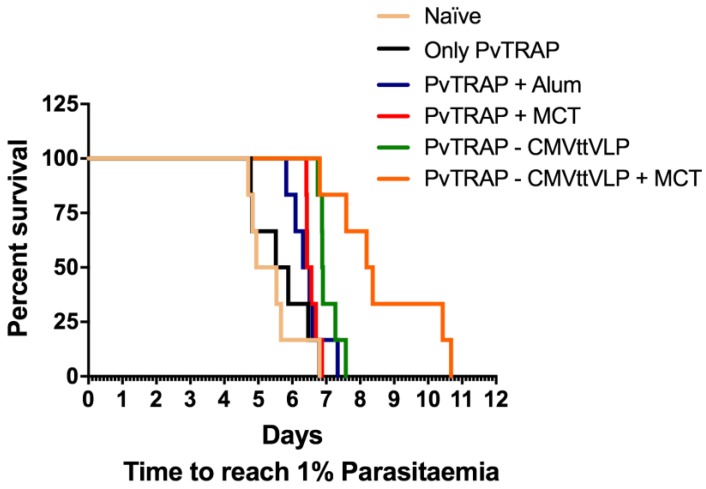
Survival curve of challenge with *P. berghei* expressing TRAP *P*. *vivax*. As previously described, the mice were challenged on Day 42 and the parasitaemia was checked daily, beginning on fourth day after challenge until the mice reach 1% parasitaemia.

One of the most efficient ways to analyse vaccine efficacy in animal models is by assessing the survival after parasite challenge. For this reason, both vaccinated and naïve (control) mice were challenged with *P. berghei* expressing *P. vivax* TRAP, as previously described [27]. The reason for using *P. berghei* instead of *P. vivax* is that the latter parasite does not infect mice, while *P. berghei* does [50,51]. As a read-out, we analysed the time until the stage 1% parasitaemia was achieved in blood. Once parasitaemia reaches 1%, mice have to be euthanized in accordance with the terms of the United Kingdom Home Office Animals Act Project License. This approach has been used previously and proven effective to demonstrate vaccine-induced protection [28,52]. The protection and delay to parasitaemia is shown in Figure 7 and Table 1 and corresponds to the humoral and cellular adaptive immune responses (Figure 4 and Figure 6 respectively). Indeed, the PvTRAP-CMVttVLP + MCT induces significant protective efficacy in this comparative investigation. Note that sterile protection is only achieved in this model using combinations of different live vectors [53,54,55]. As discussed above, antibody responses against TRAP have been suggested to be important for malaria protection [36,55] however, most studies report that anti-TRAP antibodies do not inhibit plasmodium sporozoite infectivity [56] and conclude that T cell immunity is crucial for protection against malaria [57,58,59,60]. As PvTRAP-CMVttVLP + MCT induced the strongest antibody and T cell responses and also conferred best protection, our data add to the discussion of the importance of anti-TRAP antibodies specifically, since induction of strong B and T cell responses correlated with protection against disease is reported.

## 4. Conclusions

In this study we assessed immunogenicity and protective capacity of PvTRAP formulated in MCT or alum or displayed on VLPs with or without MCT. Our results clearly show that PvTRAP conjugated to VLPs and formulated in MCT induces the highest antibody responses dominated by protective isotypes, and the highest T cell responses as well as the best protection.

It is important to consider whether a single immunogen is sufficient to induce effective protection against complex pathogens such as the malaria parasite. The life cycle of malaria parasites is particularly complex and involves different stages morphologically and antigenically. For these reasons, for a malaria candidate vaccine to be effective, it may require inclusion of a complex of antigens or multiple immunogens. However, it is important to test each antigen individually to assess its importance for applicability in any model of vaccine formulation. This is the case in our strategy; we conjugate PvTRAP to VLP and combine with MCT to show that this strategy is promising for the development of protective malaria vaccines. Of particular note is the conferred immunological synergy of using MCT and VLP. It is hypothesised that MCT offers a distinct mode of adsorption [30] and immunological profile that presents synergy with Th1 adjuvants [61] that warrants further exploration in this and other vaccine targets where the mix-/match- combination of new adjuvants are sought. Further work requires testing of alternative malaria antigens in conjunction with PvTRAP plus MCT and VLP to achieve a protection against the parasite.

## Figures and Tables

**Table 1:** Statistical analysis results of the challenge with *P. berghei* expressing *P. vivax* TRAP. As shown in this table, all groups vaccinated with protein plus adjuvant and/or VLP show significantly increased survival compared to the group vaccinated with PBS (naïve). However, when we compare naïve vs. only protein, there is no statistical difference. When comparing mice vaccinated with only protein with protein plus alum adjuvants, there was no statistical difference in survival, but if those groups are compared with MCT and or VLP, there is a significant difference in survival. The group vaccinated with PvTRAP-CMVttVLP + MCT adjuvant exhibits significantly better protection compared with all other groups. Data of six mice per group were analysed using GraphPad Prism software applied to assess the means of six groups by Tukey’s multiple comparison test of one-way ANOVA. ns = not significant, *** = *p* value < 0.0001; ** = *p* value < 0.001; * = *p* value < 0.01.

Tukey‘s Multiple Comparison Test	Significant Summary
Naïve vs. Only PvTRAP	ns
Naïve vs. PvTRAP + Alum	*
Naïve vs. PvTRAP + MCT	**
Naïve vs. PvTRAP-CMVttVLP	***
Naïve vs. PvTRAP-CMVttVLP + MCT	***
Only PvTRAP vs. PvTRAP + Alum	ns
Only PvTRAP vs. PvTRAP + MCT	*
Only PvTRAP vs. PvTRAP-CMVttVLP	**
Only PvTRAP vs. PvTRAP-CMVttVLP + MCT	**
PvTRAP + Alum vs. PvTRAP + MCT	ns
PvTRAP + Alum vs. PvTRAP-CMVttVLP	**
PvTRAP + Alum vs. PvTRAP-CMVttVLP + MCT	***
PvTRAP+MCT vs. PvTRAP-CMVttVLP	*
PvTRAP+MCT vs. PvTRAP-CMVttVLP + MCT	**
PvTRAP-CMVttVLP vs. PvTRAP-CMVttVLP + MCT	*
